# Measuring naturally acquired *ex vivo* IFN-γ responses to *Plasmodium falciparum* cell-traversal protein for ookinetes and sporozoites (CelTOS) in Ghanaian adults

**DOI:** 10.1186/s12936-014-0539-5

**Published:** 2015-01-21

**Authors:** Dorothy Anum, Kwadwo A Kusi, Harini Ganeshan, Michael R Hollingdale, Michael F Ofori, Kwadwo A Koram, Ben A Gyan, Susan Adu-Amankwah, Edem Badji, Jun Huang, Maria Belmonte, Glenna J Banania, Theophilus B Kwofie, Eileen Villasante, Daniel Dodoo, Martha Sedegah

**Affiliations:** Kwame Nkrumah University of Science and Technology, Kumasi, Ghana; Noguchi Memorial Institute for Medical Research, University of Ghana, Legon, Ghana; US Military Malaria Vaccine Program, Naval Medical Research Center, Maryland, USA

**Keywords:** T cell, *ex vivo* IFN-γ ELISpot, Malaria, Immune response, Peptides

## Abstract

**Background:**

A malaria vaccine that targets the sporozoite/liver stage parasites could potentially prevent blood stage infection and the associated clinical symptoms. Identification of sporozoite/liver stage antigens is, therefore, crucial for the development of effective vaccines. Cell-traversal protein for ookinetes and sporozoites (CelTOS) is a highly conserved antigen involved in sporozoite motility and hepatocyte invasion and has been shown to induce significant IFN-γ production in PBMCs from radiation-attenuated sporozoite-immunized malaria-naïve individuals. The aim of this study was to ascertain whether such CelTOS-specific recall responses are also induced in individuals with natural exposure to *Plasmodium falciparum*.

**Methods:**

*Ex vivo* IFN-γ responses to 15mer overlapping peptide pools covering the entire sequence of CelTOS and five other candidate antigens, CSP, AMA1, MSP1, TRAP and LSA1, were characterized using PBMCs from 35 malaria exposed adults. Responses to four CelTOS peptide pools (CelTp1, CelTp2, CelTp3 and CelTp4), a pool containing peptides from the entire CelTOS antigen (CelTTp), and pools comprised of overlapping peptides from each of the other five malaria antigens were assessed by *ex vivo* ELISpot assay. A positive IFN-γ response for stimulants was defined by two criteria; a stimulation index of two or greater relative to the unstimulated control, and a difference of 10 or greater in spot forming cells between stimulant and the unstimulated control.

**Results:**

Of the 35 volunteers tested, five had positive IFN-γ recall responses against the four different CelTOS pools while four volunteers made responses against the CelTTp pool; six volunteers were, therefore, positive with CelTOS. By contrast, six volunteers responded to AMA1, seven to LSA1, 15 to MSP1 and two volunteers responded against CSP and TRAP.

**Conclusions:**

These results suggest natural malaria transmission induces CelTOS-specific *ex vivo* IFN-γ in Ghanaian adults and that the frequency of these responses was similar to those of other previously characterized malaria antigens. These findings support the further evaluation of CelTOS as a pre-erythrocytic candidate antigen for inclusion in a potential multi-antigen vaccine.

**Electronic supplementary material:**

The online version of this article (doi:10.1186/s12936-014-0539-5) contains supplementary material, which is available to authorized users.

## Background

Current malaria control strategies combine identification and elimination of vectors with prompt diagnosis and treatment of infected individuals in endemic populations. Various insecticides have been applied for the control of the mosquito vectors, and chemoprophylaxis is used to prevent blood stage infection associated with the clinical symptoms of malaria. These disease control measures are however being hampered by the resistance of parasites and mosquito vectors to drugs and insecticides respectively [1]. Vaccines are an essential and cost-effective public health tool and it is believed that the development of anti-malarial vaccines would be an important addition to existing control strategies. The feasibility of developing a malaria vaccine is firstly suggested by the acquisition of partial clinical immunity following repeated exposure to parasites during natural transmission in malaria-endemic areas [2,3]. Secondly immunization with radiation-attenuated sporozoites has been shown to induce sterile protection against the sporozoite and liver stages of the parasite [4,5].

The malaria parasite has a complex life cycle and it is believed that an effective anti-malarial subunit vaccine may need to target antigens in multiple stages of the parasite. The *Plasmodium falciparum* cell-traversal protein for ookinetes and sporozoites (CelTOS) is required for motility of the parasite in both the mosquito vector and the human host, and is required for successful malaria infections [6]. Anti-CelTOS antibody responses in mice have been shown to inhibit sporozoite motility and invasion of hepatocytes *in vitro,* and induced sterile protection in test animals [7,8]. Moreover, CelTOS peptides elicited proliferative and IFN-γ responses in *ex vivo* ELISpot assays using peripheral blood mononuclear cells (PBMCs) from irradiated sporozoite-immunized volunteers [9]. There are, however, no reports on the induction of naturally acquired cell-mediated immune responses to CelTOS in populations in malaria-endemic areas.

E*x vivo* ELISpot assays have previously been shown to be capable of detecting recall IFN-γ responses from adults in Ghana using HLA-matched DR- or class I-restricted peptides derived from the circumsporozoite protein (CSP), thrombospondin-related adhesion protein (TRAP), liver stage antigen-1 (LSA1), liver stage antigen-3 (LSA3) and exported protein-1 (EXP1) [10]. Moreover, since responses were generally low, in the same study non-HLA matched peptides from CSP and apical membrane antigen-1 (AMA1) were used to determine the reproducibility of *ex vivo* ELISpot assays, and found that using positivity criteria defined as at least a two-fold difference comparing test samples and medium controls, and at least a difference of 10 spot forming cells (sfc) per million PBMC between test samples and medium controls, led to 60% reproducibility [10]. The aim of this study was, therefore, to determine whether CelTOS peptides could induce IFN-γ recall responses in PBMCs from volunteers in Ghana using *ex vivo* ELISpot assays, using the positivity criteria based on that study [10]. Pools of peptides from CSP, TRAP, LSA1, AMA1 and merozoite surface protein-1 (MSP1) were concurrently used for PBMC stimulation. For each of these additional antigens, a single pool of peptides covering the entire antigen was used, rather than HLA-matched individual peptides, with the assumption that such pools contain multiple HLA-restricted epitopes that match the HLA of each subject. The results showed that with the exception of MSP1, CelTOS peptides recalled a similar frequency of positive responses as the other four antigens, suggesting that CelTOS may be a potentially important antigen for inclusion in a multi-antigen malaria vaccine.

## Methods

### Ethics

This study was conducted according to a human use protocol “Quality Control of Immunological Reagents and Validation of Improvements to Immunological Assays in Support of Malaria Vaccine Trials” approved by Intuitional Review Boards at the Noguchi Memorial Institute for medical Research (NMIMR) and the Naval Medical Research Center (NMRC). NMIMR holds a United States Government Federal Wide Assurance (FWAA00001824) from the Office for Human Research Protections, as does NMRC (FWA00000152). NMRC also holds a Department of Navy Addendum to the FWA for human subject protections. Written informed consent was sought from all study participants who willingly agreed to be part of the study and met the inclusion criteria.

### Study area and sampling

The study was conducted within the University of Ghana, Legon and its surrounding communities in Accra, Ghana. Legon is an urban community that lies within latitude 5.65 (5° 39′ 0 N) and longitude −0.18 (0° -11' 0 W). It is about 10 kilometres north from the capital city, Accra. It is home to the University of Ghana, and a 10 square km area around Legon has an approximate population of 100,000. The average annual rainfall in the study area is below 1000 mm and more than half of this figure is usually recorded between April and June. Malaria transmission follows the pattern of rainfall and most malaria admissions to the health facilities occur between May and August. Malaria transmission in the study area however occurs mainly along the peri-urban fringes that have suitable breeding sites for the mosquito vector. Malaria slide positivity in the study area is usually below 1% for most of the year [10]. Study participants were male and female adults between 24–43 years (average age 29 years) who were resident in the study area. Female participants were neither pregnant nor nursing. Urine samples of female study participants were tested using test strip (Accurate Pregnancy Urine Test kit, USA) for the detection of human chorionic gonadotrophin (hCG). The level of haemoglobin (Hb) in study participants was measured using HemoCue (HemoCue® Hb 201 System, Sweden). Participants with haemoglobin > 10 g/dl and whose blood pressure fell outside the range 120-139/80-89 were excluded from the study. All participants generally had a normal medical history at screening and physical examination. A total of 45 volunteers were screened and 35 who met the inclusion criteria were included in the study. The 35 selected study participants were subsequently screened for malaria parasites by rapid diagnostic test (RDT) kits and by light microscopy. Sixty millilitres (60 ml) of venous blood was collected per participant into heparinized tubes. PBMCs were isolated from blood by gradient centrifugation using Accuspin Histopaque-1077 cell separating tubes. After washing and counting, cells were rested in an incubator at 37°C, 5% CO_2_ for a maximum of 20 h before use in *ex vivo* ELISpot assays.

### Synthetic peptides and peptide pools

Synthetic 15mer peptides that overlap by 11 amino acids and span the full length CelTOS, CSP, AMA1, TRAP, the 42 kDa fragment of MSP1 and truncated C-terminal portion of LSA1, all 3D7 strain, were synthesized by Mimotopes, VIC, Australia (>80% purity). CelTOS peptides were grouped into four CelTOS peptide pools (CelTp1, CelTp2, CelTp3 and CelTp4) or combined into one pool (CelTTp) (Table 1). Similarly, overlapping 15mer peptides that represent either the full length antigens (CSP, TRAP, AMA1) or partial antigen sequences (LSA1 and MSP1) were combined into single peptide pools per antigen, designated as CSPp, AMA1p, LSA1p, TRAPp and MSP1p.

**Table 1:** **CelTOS peptide pools used in ELISpot assays**

**Peptide pool**	**Peptide pool name**	**Amino acids**	**Number of peptides**
CelTOS pool 1	CelTp1	1 - 55	11 peptides (1–11)
CelTOS pool 2	CelTp2	45 - 99	11 peptides (12 – 22)
CelTOS pool 3	CelTp3	89 - 143	11 peptides (23 – 33)
CelTOS pool 4	CelTp4	133 - 182	10 peptides (34 – 43)
CelTOS single pool	CelTTp	1 - 182	43

### *Ex vivo* ELISpot IFN-γ Assay

ELISpot IFN-γ assays were performed as previously described [10]. Multiscreen plates (Millipore Corporation, USA) were coated with Monkey anti-human IFN-γ (Mabtech AB, USA) and PBMCs (400,000 cells/well) from each volunteer were tested in duplicate with 10 μg/ml of each of the five CelTOS peptide pools (CelTp1, CelTp2, CelTp3, CelTp4, CelTTp) or with 1.25 μg/ml of the other single pools from CSPp, AMA1p, LSA1p, TRAPp, and MSP1p. Concanavalin A (Con A, Sigma Aldrich, USA) at a concentration of 0.313 μg/ml and CEF (consisting of 32 HLA class I-restricted peptides from CMV, EBV and Flu, Cellular Technology Ltd, USA.) at a concentration of 2.0 μg/ml were used as positive control stimulants and tested in triplicate with 100,000 PBMCs/well as previous data showed that for most volunteers, testing at 400,000 PBMCs per well gave spots that were too numerous to count [11]. Volunteer PBMCs that were incubated with medium only were used as controls and PBMCs from malaria-naive volunteers from USA were incubated with all test peptides and CEF for internal control purposes. After PBMC incubation for 36 hours, spots were detected by incubation with biotinylated anti-IFN-γ polyclonal antibody (Mabtech, USA) and subsequently with alkaline-phosphatase-conjugated streptavidin (Mabtech, USA). After plate development with chromogenic substrate for alkaline phosphatase (Bio-Rad, USA), the number of IFN-γ-producing spots per well was estimated using an automated ELISpot plate reader (AID GmbH, Germany) and the acquired data was exported into Microsoft Excel for analysis.

### Data analysis

Activities were calculated as spot forming cells per million PBMCs (sfc/m). The assay was considered positive if there was (1) at least a doubling of sfc/m in test wells relative to control wells, and (2) a difference of at least 10 spots between test and control wells, based on our previous studies [10]. A volunteer was considered positive to a malaria antigen if his/her PBMC tested positive against at least one peptide pool. Fisher’s exact test was used to compare proportions of IFN-γ responders between the CelTTp pool and those of the five other *P. falciparum* antigens (CSP, AMA1, TRAP, LSA1 and MSP1). Subsequently a pairwise post hoc test for differences in proportions was performed when statistically significant differences were observed in proportions of positive responders to the various antigens. Statistical analysis and graphics were done with Graph Pad prism (version 5.04, San Diego, CA, USA) and the R statistical package (version 3.0.2, R development core team). A p value less than 0.05 was considered statistically significant.

## Results

### Participant flow

A total of 45 healthy Ghanaian adults were screened and 35 volunteers who met eligibility requirements and gave informed consent participated in the study. Volunteers were between 24 and 43 years with an average age of 29 years. All female volunteers tested negative for pregnancy and all volunteers were negative for malaria parasitaemia by light microscopy and malaria RDT. All volunteers made positive IFN-γ responses to Con A or CEF or both (Additional file 1). For each volunteer, the ELISpot activity (sfc/m) for the unstimulated medium control was subtracted from the activities (sfc/m) for each test peptide. In all assays, unstimulated medium control responses ranged between 0 and 18 sfc/m except volunteers v15 and v28 whose mean unstimulated medium responses were 69 and 88 sfc/m respectively.

### *Ex vivo* ELISpot IFN-γ responses to CelTOS peptide pools

**Figure 1:**
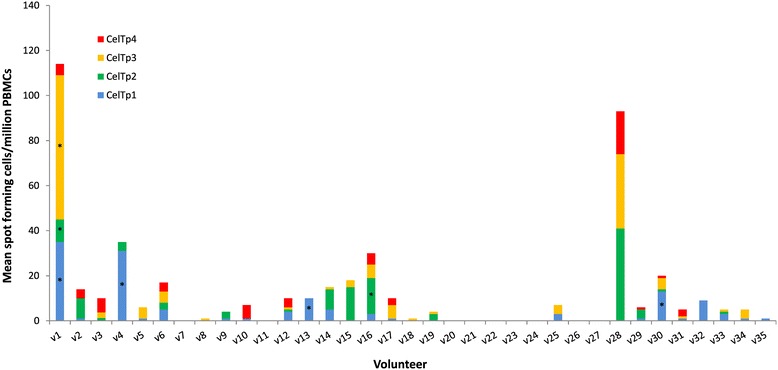
**Magnitude of IFN- γ responses to the four separate CelTOS pools.** For each volunteer, stacked bars represent responses to the five pools and bars with asterisks (*) are responses that were positive based on the set positivity criteria. The plotted data are those over the medium background responses (difference between activities for test peptide-stimulated PBMCs and unstimulated control PBMCs).

**Figure 2:**
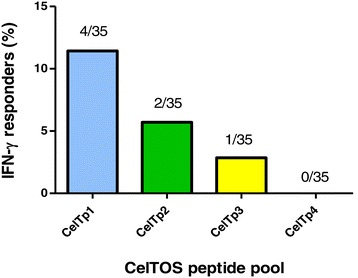
**Proportion of IFN-γ positive responders to the four separate CelTOS pools.** The absolute number of responders for each pool has been expressed as a proportion of the total number of volunteers (35).

The magnitude of IFN-γ responses (sfc/m) to the CelTOS 15mer peptide pools CelTp1, CelTp2, CelTp3 and CelTp4 is shown in Figure 1, and the proportion of volunteers that responded to each pool is presented in Figure 2. Overall, five of the 35 (14%) study volunteers (v1, v4, v13, v16 and v30) were positive to at least one of the CelTOS peptide pools. Volunteer v28 had high activities in both test and control wells and was therefore not considered positive. Four of the five positive volunteers had positive activities to CelTp1 (v1, v4, v13 and v30), two of these were also positive with CelTp2 (v1 and v16) and one (v1) was positive with CelTp3. No activity was detected to CelTp4. The highest response was that of volunteer v1 to CelTp3 (64 sfc/m) and this volunteer also had the most positive responses (against CelTp1, CelTp2 and CelTp3, Figure 1). The remaining 30 volunteers did not respond to any of the CelTOS peptide pools, although they responded positively to Con A and CEF, except for v26 who responded only to CEF (Additional file 1). PBMCs from two malaria-naïve donors that were repeatedly stimulated with the same parasite peptides pools did not make any antigen-specific responses, though these cells also responded to stimulation with CEF controls (Additional file 2).

### *Ex vivo* IFN-γ responses to CelTTp, CSPp, AMA1p, LSA1p, TRAPp and MSP1p pools

**Figure 3:**
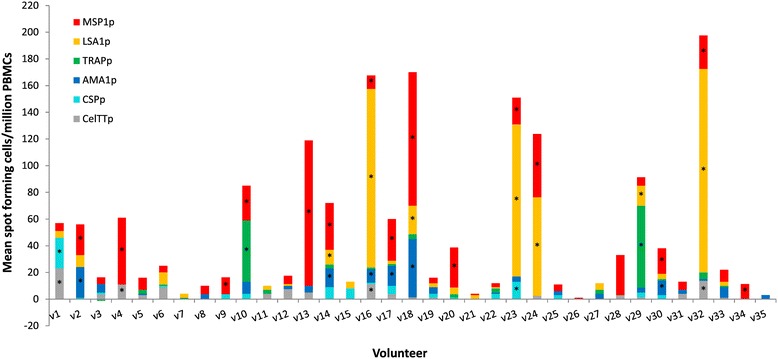
**Magnitude of IFN- γ responses to CelTTp and the five other malarial antigens.** For each volunteer, stacked bars represent responses to the six single pools and bars with asterisks (*) are positive responses as defined in the Methods. The plotted data are those over the medium background responses (difference between activities for test peptide-stimulated PBMCs and unstimulated control PBMCs).

**Figure 4:**
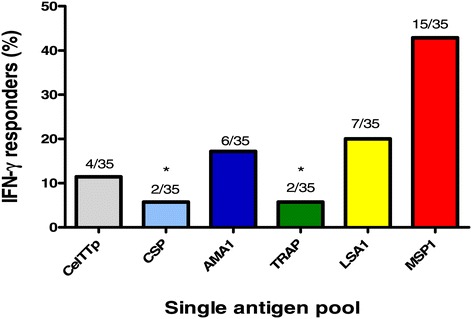
**Proportion of IFN-γ positive responders to the six malaria vaccine candidate antigens.** The absolute number of responders for each pool has been expressed as a proportion of the total number of volunteers (35). *Proportions that were positive against CSPp and TRAPp were significantly different from that of MSP1p in pairwise comparisons.

IFN-γ responses to peptide pools representing the six tested malaria antigens CelTOS, CSP, AMA1, LSA1, TRAP and MSP1 are shown in Figure 3. Responses differed among volunteers, probably due to their different HLA molecules. However, 17 of the 35 volunteers (49%) responded to at least one of the tested malaria antigen peptide pools. The frequency of positive volunteers to each antigen is shown in Figure 4. The most frequent positive response was to MSP1p (15/35 volunteers, 43%), followed by LSA1p (7/35, 20%), AMA1p (6/35, 17%), CelTTp (4/35, 11%), CSPp (2/35, 6%) and TRAPp (2/35, 6%). Three of the five volunteers that were positive against at least one of the four separate CelTOS pools (CelTp1, CelTp2, CelTp3 and CelTp4) were also positive against CelTTp (v1, v4, and v16), and in addition one volunteer (v32) who was negative against all four separate CelTOS pools was positive against CelTTp. Overall, among the 35 volunteers, four/35 (11%) were positive against one antigen (only MSP1p), 13/35 (29%) were positive against at least two different antigens, five/35 (14%) were positive against at least three antigens, and only v16 (3%) was positive against four antigens. Six of thirty five (6/35) volunteers (17%) had summed activities greater than 100 sfc/m, and LSA1p and/or MSP1p made the greatest contributions; TRAPp made the greatest contributions to the next two highest volunteers (6%). Eighteen volunteers (51%) did not respond to any of the malaria antigen pools although they responded to stimulation with Con A and CEF (Additional file 1).

### Comparison of CelTOS peptide pool responses to those of other anti-malarial antigen pools

Comparison of responder frequencies to peptide pools from the six malaria antigens tested showed statistical significance in differences between at least two of the six peptide pool responses (p = 0.0006, Fisher’s Exact test). Post-Hoc pairwise comparison revealed that the proportion of positive responses against CelTTp was not significantly different from that against any of the other five antigen pools (p > 0.05 in all cases, pairwise proportion tests with Holm’s correction, Table 2). The proportion of positive responses against MSP1p was however significantly higher than those against TRAPp and CSPp (p = 0.012 in both cases, Table 2). Comparison of the median activities (sfc/m) to each peptide pool for the 35 volunteers showed that there were no statistically significant differences in activities amongst the antigens (p = 0.27, Kruskal-Wallis test). Therefore, the proportion of positive volunteers and the magnitude of responses to CelTTp were similar to those observed for the malaria vaccine candidates CSP, TRAP, LSA1, and AMA1 and MSP1.

**Table 2:** **P values for pairwise comparison of responder proportions amongst single antigen pools**

	**AMA1p**	**CelTTp**	**CSPp**	**LSA1p**	**MSP1p**
CelTTp	1.000	-	-	-	-
CSPp	1.000	1.000	-	-	-
LSA1p	1.000	1.000	1.000	-	-
MSP1p	0.443	0.094	0.012	0.787	-
TRAPp	1.000	1.000	1.000	1.000	0.012

Pairwise comparison of responder frequencies to all antigens was performed with Holm’s correction. Prior to performing the pairwise test, Fisher’s Exact test for multiple comparison of proportions of positive volunteers to the six peptide pools showed that the proportion of positive responders was significantly different between at least two pairs of antigens (p < 0.05). Proportions of positive responders for antigen pools are shown in Figure 4.

## Discussion

The development of an effective anti-malarial vaccine is an important public health priority as it will add to the currently available disease control tools. Subunit vaccine development requires the identification of conserved immunodominant antigens from multiple stages of the parasite in order to ensure broad protection and curtail disease transmission. CelTOS is a pre-erythrocytic malaria vaccine candidate antigen that has been shown to induce potent antibody and T cell responses and prevent the establishment of blood stage infection in mice [6-8]. CelTOS 15mer peptides also elicited HLA-restricted IFN-γ responses in *ex vivo* ELISpot assays using PBMC from irradiated sporozoite-immunized volunteers [9]. Moreover, CelTOS is a relatively conserved antigen and elicits cross-protective immune responses against heterologous challenge with *Plasmodium berghei* [6,7]. CelTOS based vaccines have been shown to elicit both antibody and T cell responses in experimental animal models [7,8,12,13], and phase 1 clinical trials of a CelTOS vaccine known as FMP012 in malaria-naïve volunteers has just been completed (https://clinicaltrials.gov/ct2/show/NCT01540474). However, very little is known about naturally induced immune responses to CelTOS in a population with exposure to *P. falciparum*. A previous study with volunteers aged one to 30 years and living in a low malaria transmission area of southern Ghana showed very low levels of naturally induced CelTOS-specific antibodies [14]. The aim of the current study was to investigate the induction of CelTOS-specific IFN-γ recall responses in PBMCs from Ghanaian adult volunteers in an area (Legon) with natural exposure to *P. falciparum* parasites, which was previously confirmed by seropositivity to erythrocytic stage antigens [10]. However, in that previous study, only a proportion of subjects had recall ELISpot activities to six malaria antigens that were tested, suggesting that cellular responses may be shorter-lived than antibody responses [10].

Four pools of 15mer peptides that together span the entire amino acid sequence of CelTOS were tested against PBMCs from 35 study volunteers. Fourteen percent (14%) of volunteers made positive responses to the four CelTOS peptide pools, and all positive responses were to the first three peptide pools (CelTp1, CelTp2 and CelTp3) with no response to CelTp4 (Figures 1 and 2). By contrast, 49% of volunteers made positive responses to one or more of the six single peptide pools spanning the full length antigens (CelTTp, AMA1p, CSPp and TRAPp) or partial sequences (LSA1p and MSP1p), shown in Figures 3 and 4. Since one volunteer who was negative with the individual CelTOS pools, was positive to the single CelTTp pool, six/35 (17%) of volunteers were positive to CelTOS. This was not related to the viability of the PBMC, as all volunteers made positive responses to the Con A and CEF (Additional file 1), with the exception of volunteers v13, v16 and v26 whose cells at 100,000/well did not make responses to Con A, suggesting that PBMCs used for IFN-γ response assessment were viable. The magnitude of responses to CEF peptides in this study (Additional file 1) was generally similar to those in a previous study with volunteers from the same study area [10]. The mean CEF response in PBMCs from the 29 volunteers with positive IFN-γ responses (1032 sfc/m) was however up to five times higher than the average response (216 sfc//m) measured in PBMCs from the two malaria-naïve volunteers (Additional file 2). In addition, anti-CelTOS IFN-γ responses observed in this study were several orders lower than the average response observed in a previous study with PBMCs from attenuated sporozoite-immunized volunteers [9].

These results show that peptide pools from the N-terminal end of CelTOS (CelTp1, CelTp2, CelTp3) induced responses whereas the C-terminal peptide pool (CelTp4) did not (Figure 1). This suggests a greater prevalence of T cell epitopes in the N-terminal region of CelTOS. Preliminary predictions using the bioinformatic algorithm NetMHC [15] identified more Class 1-restricted epitopes within CelTp1, CelTp2 and CelTp3 than CelTp4 [9]. This is in agreement with the *ex vivo* ELISpot results presented here and suggest that at least some of the responses observed in the current study are likely to be CD8+ T cell-specific. Despite the conserved nature of the *celtos* gene, a limited number of single nucleotide polymorphisms (SNPs) have been mapped to the C-terminal region of the protein sequence that has been predicted to have immunodominant B cell epitopes [16] and may thus be more involved in immune escape against CelTOS-specific antibody responses. *Ex vivo* ELISpot analysis of mouse PBMCs showed a concentration of immunogenic T cell epitopes at the C terminal end of CelTOS, although murine HLA-restricted CD4+ and CD8+ epitopes were predicted by bioinformatics tools to be distributed over the entire antigen in the same study [17]. It is possible that differences between murine and human HLA-restricted CelTOS epitopes may explain differences in ELISpot data between that study and the current study. However, on the basis of data from this and other human studies [9], there is likely to be little effect of the limited polymorphism in CelTOS on anti-CelTOS T cell IFN-γ responses. Elucidation of the HLA restriction of observed responses by CD4+/CD8+ cell depletion ELISpot assays or by flow cytometric analysis and HLA-specific peptides will help determine the T cell subset specificity of observed responses and further establish the use of CelTOS as a malaria vaccine in genetically-diverse populations especially in Africa [18].

In these studies the proportion of positive volunteers was greatest to the blood stage antigen MSP1 but statistically, only the difference between responders to MSP1 on the one hand and CSP and TRAP on the other hand was significant (Figure 4, Table 2). The proportions of positive volunteers to the other antigens were statistically similar. The overall magnitudes of responses were similar against all antigens tested; however, individual responses were highest against MSP1, LSA1 and TRAP in eight volunteers (Figure 3). These observations may be partially explained by the fact that blood stages persist for longer during malaria infection and thus induce responses in a greater proportion of volunteers (MSP1), while LSA1 contains more immunogenic epitopes than other malaria antigens [19]. A number of studies in both naturally exposed individuals and naïve volunteers immunized with radiation-attenuated sporozoites have found numerous CD4+ and CD8+ T cell epitopes in MSP1, with most of these being limited especially to the 42 kDa fragment of protein [20-22]. However, the involvement of CD4+ or CD8+ T cells in these ELISpot activities was not determined in the current study.

In a previous study, short Class I-restricted and DR-restricted long peptides representing CSP, TRAP, and LSA1 were used as stimulants and these matched the HLA of volunteers in Ghana [10]; in total 12/26 volunteers (46%) were positive, comparable with the total number 17/35 volunteers (49%) who were positive against CSP, TRAP and LSA1, in addition to AMA1 and MSP1 in this study (Figure 3). Thus pools of peptides spanning the entire length of malaria antigens were as effective in eliciting *ex vivo* IFN-γ responses as HLA-matched single peptides. In that earlier study the highest frequency of responses was to LSA1 [10] and MSP1 was not tested in that earlier study.

In summary, CelTOS-specific T cell responses were detected in five out of 35 study volunteers who have a history of exposure to *P. falciparum*. The proportion of volunteers who responded to the CelTOS antigen was not significantly different from the proportions that responded to other established malaria vaccine candidate antigens. Immune responses to CelTOS may block the development of pre-erythrocytic parasites and have the potential to protect malaria endemic populations by reducing or preventing infection of liver cells by sporozoites, thereby reducing disease severity. Since natural transmission and immunization with irradiated sporozoites induce cellular IFN-γ responses to CelTOS, these findings support further evaluation of CelTOS as a pre-erythrocytic candidate antigen for inclusion in a potential multi-antigen vaccine. It has been shown previously that two vaccine candidate antigens, CSP and AMA1, combined in a DNA-prime, adenovirus-boost vaccine regimen induced complete protection that was primarily mediated by CD8+ T cell IFN-γ responses in 4/15 volunteers to malaria challenge [23,24]. It is possible that inclusion of other antigens such as CelTOS that also induce cellular IFN-γ responses may increase efficacy of this vaccine.

## Conclusions

Natural malaria transmission in an endemic area in Ghana induces cellular IFN-γ responses against CelTOS in adults that were similar to responses against other pre-erythrocytic antigens CSP, TRAP and LSA1. These results support further development of CelTOS as a malaria vaccine, either alone or in combination with other antigens.

## Additional files

Additional file 1:
**IFN-γ responses to Con A and CEF in PBMCs from the 35 volunteers.** One hundred thousand (100,000) PBMCs were stimulated in triplicate with both Con A and the CEF peptide pool and the resulting number of spots averaged and expressed as sfc/m PBMCs. The data plotted are responses over the medium background responses. Volunteers v1, v6, v18, v19, v23 and v33 were positive to Con A but not to CEF while v13, v16 and v26 were positive to CEF but not to Con A, based on the set positivity criteria. Responses by all other volunteers were positive to both Con A and CEF. Additional file 2:
**IFN-γ responses to all test peptides and CEF in PBMCs from two malaria-naïve volunteers.** Test peptides were used to stimulate 400,000 PBMCs/well and CEF was used to stimulate 100,000 PBMCs/well of malaria-naïve volunteer PBMCs before expression as sfc/m PBMCs. Responses in both volunteers were negative to all test peptides and positive to the CEF peptide pool.
