# Supplementary figures and images for: Measuring naturally acquired ex vivo IFN-γ responses to Plasmodium falciparum cell-traversal protein for ookinetes and sporozoites (CelTOS) in Ghanaian adults
Source: Malar J. 2015 Jan 21;14:20. doi: 10.1186/s12936-014-0539-5 (PMC4308902; doi:10.1186/s12936-014-0539-5)

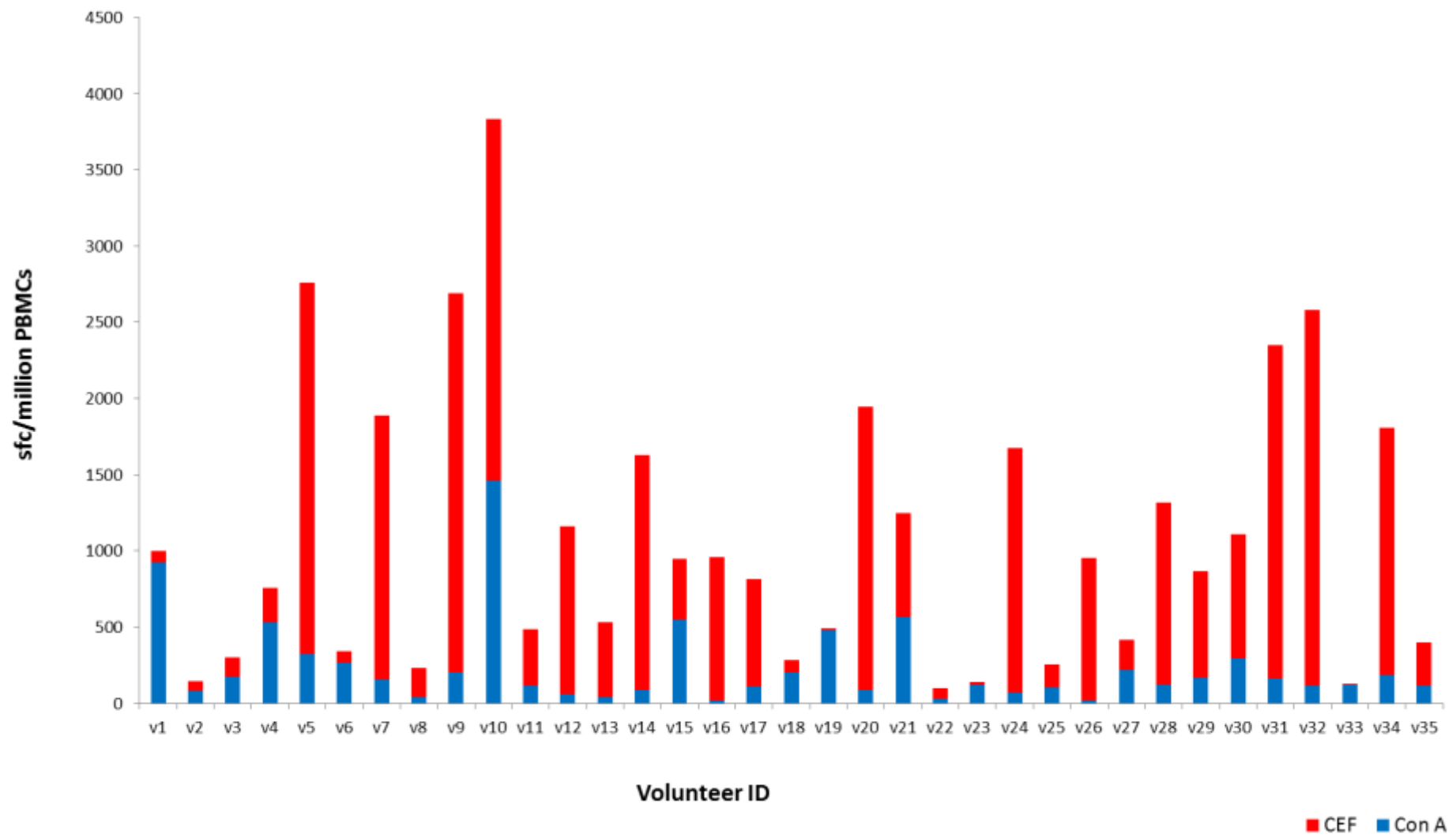

Supplement: Additional file 1: — IFN-γ responses to Con A and CEF in PBMCs from the 35 volunteers. One hundred thousand (100,000) PBMCs were stimulated in triplicate with both Con A and the CEF peptide pool and the resulting number of spots averaged and expressed as sfc/m PBMCs. The data plotted are responses over the medium background responses. Volunteers v1, v6, v18, v19, v23 and v33 were positive to Con A but not to CEF while v13, v16 and v26 were positive to CEF but not to Con A, based on the set positivity criteria. Responses by all other volunteers were positive to both Con A and CEF. [file 12936_2014_539_MOESM1_ESM.pdf]

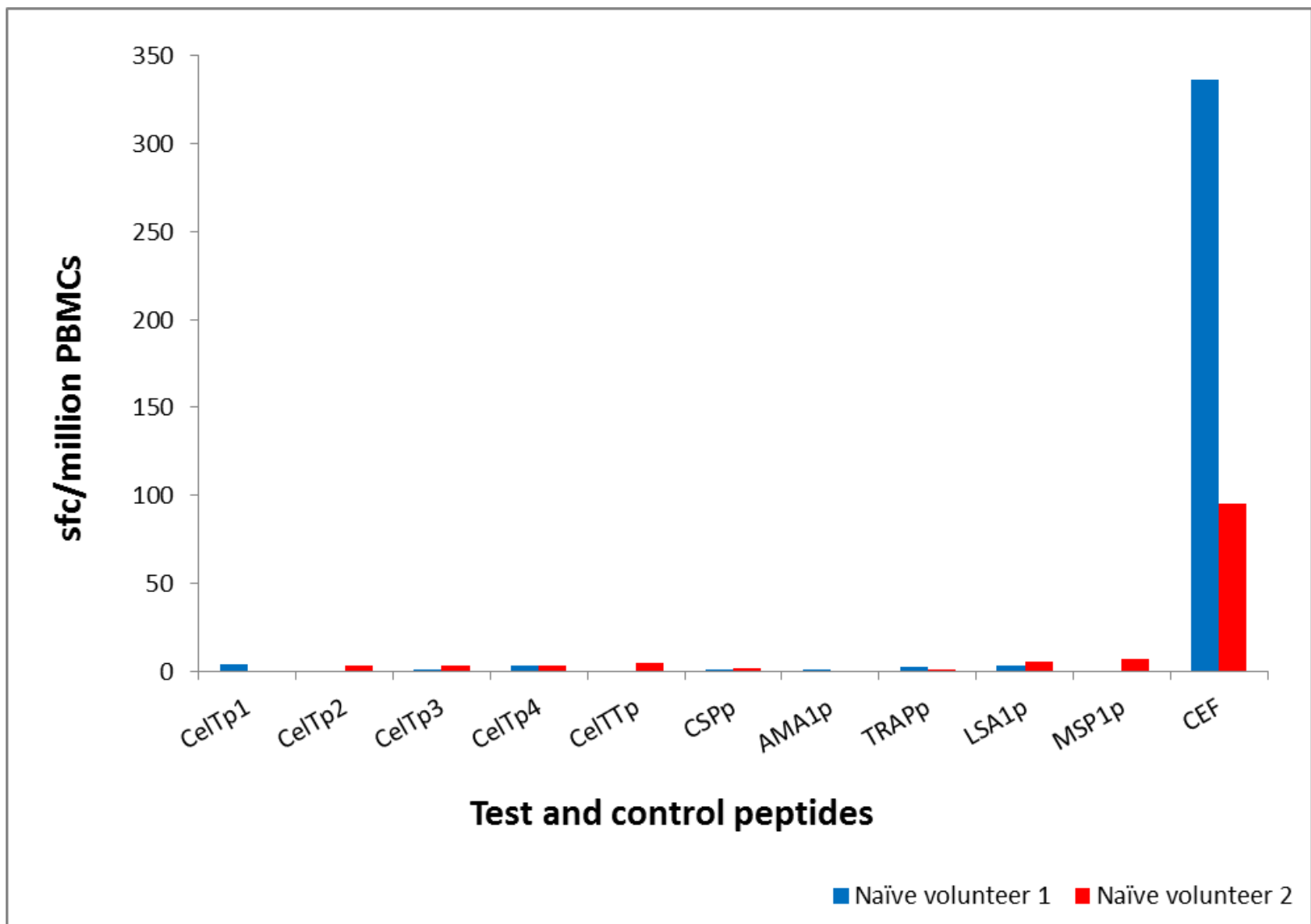

Supplement: Additional file 2: — IFN-γ responses to all test peptides and CEF in PBMCs from two malaria-naïve volunteers. Test peptides were used to stimulate 400,000 PBMCs/well and CEF was used to stimulate 100,000 PBMCs/well of malaria-naïve volunteer PBMCs before expression as sfc/m PBMCs. Responses in both volunteers were negative to all test peptides and positive to the CEF peptide pool. [file 12936_2014_539_MOESM2_ESM.pdf]
